# Celebrating 70 years of DNA discovery: exploring the Blueprint of Life

**DOI:** 10.25122/jml-2024-1007

**Published:** 2024-04

**Authors:** Alexandru Vlad Ciurea, Luca-Andrei Glavan, Horia Petre Costin, Razvan-Adrian Covache-Busuioc, Felix Mircea Brehar

**Affiliations:** 1 Department of Neurosurgery, ‘Carol Davila’ University of Medicine and Pharmacy, Bucharest, Romania; 2 Neurosurgery Department and Scientific Director, Sanador Clinical Hospital, Bucharest, Romania; 3 Romanian Academy; 4 Department of General Medicine, ‘Carol Davila’ University of Medicine and Pharmacy, Bucharest, Romania; 5 Department of Neurosurgery, ‘Carol Davila’ University of Medicine and Pharmacy, Bucharest, Romania; 6 Neurosurgery Department, ‘Bagdasar-Arseni’ Emergency Hospital, Bucharest, Romania

## INTRODUCTION

The history of genetics can be studied in many ways, each offering valuable insights. An especially fruitful approach is to follow the gradual emergence of our understanding of the nature of genetic material. This method holds particular relevance as we commemorate 60 years since the groundbreaking discovery of the molecular structure of deoxyribonucleic acid (DNA) by James D. Watson and Francis H.C. Crick [1].

Since its original conception in 1953, the DNA model has withstood rigorous scrutiny through physicochemical and electron microscopic studies, confirming its validity. Indeed, its double-helix shape has transcended biology, becoming an icon of scientific knowledge and a cultural symbol in our modern age.

After 18 months of tireless effort, Watson, then 24, and Crick, then 36, made their groundbreaking discovery in Cambridge, England, on 28 February 1953. Rarely is any scientific discovery so definitively marked; James D. Watson vividly captured the journey and subsequent events in his book “The Double Helix” [2].

Horace F. Judson suggests that the initial publication of the DNA model on 25 April 1953, was notable for its conciseness: it included just 800 words and one figure. Judson attributes its brevity to the highly competitive environment in which the authors found themselves. Watson, in particular, saw it as an uphill race against rivals such as American chemist Linus C. Pauling and British biophysicist Rosalind E. Franklin, who were both vying for attention at that time [3,4].

After publishing their model, Watson and Crick quickly expanded upon it by providing more precise descriptions of the structure of DNA and its genetic implications [5]. Later that same year, they presented an in-depth examination of the model at the Cold Spring Harbor Symposia on Quantitative Biology [6].

The Watson–Crick Model displays remarkable ingenuity by offering an exhaustive explanation for four essential properties required of genetic material. First and foremost, the model elucidates DNA replication – an essential characteristic of life itself – facilitating reproduction. Second, it accounts for the specificity of genetic material by considering its unique traits during replication. Third, the model highlights the informative capability of DNA as a macromolecule. Finally, it clarifies the adaptability of genetic material by demonstrating its capacity for mutation. These four essential and indispensable properties will be further explored in future discussions.

Watson and Crick [1] first published their DNA model in the journal *Nature*. Subsequent X-ray crystallography studies conducted by British scientists Maurice H.F. Wilkins, Rosalind E. Franklin, and Raymond G. Gosling were later published in the same journal [7,8], providing further validation of Watson and Crick’s model. Further biophysical and electron microscopic research has since confirmed its accuracy.

**Figure 1:**
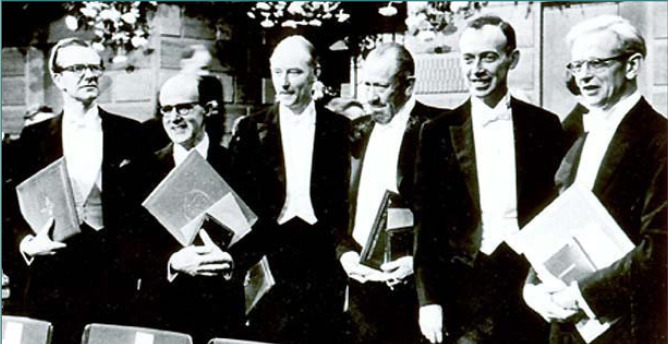
1962 Nobel Prize winners (L-R): Maurice Wilkins (Physiology or Medicine), Max Perutz (Chemistry), Francis Crick (Physiology or Medicine), John Steinbeck (Literature), James Watson (Physiology or Medicine), John Kendrew (Chemistry).

Watson, Crick, and Wilkins were honored with the Nobel Prize in Physiology or Medicine in 1962 for their groundbreaking research on the molecular structure of nucleic acids and their role in transmitting information within living organisms. Rosalind Franklin made substantial contributions to understanding the structure of DNA, but tragically, she died of ovarian cancer at the age of 37. Although her work was crucial, she was ineligible for the Nobel Prize, as it cannot be awarded posthumously or divided among more than three recipients (Figure 1).

## EARLY STAGES

In 1869, Friedrich Miescher from Basel, Switzerland, made an important discovery in genetics while working in Professor Felix Hoppe-Seyler’s laboratory at Tübingen University in Germany. Miescher conducted extensive studies of cell nuclei, using material collected from surgical bandages with pus, which contained white blood cells known as leukocytes, from local hospitals. Through extensive purification of cell nuclei, Miescher successfully isolated a novel organic substance he termed ‘nuclein’.

Nuclein distinguished itself from other cell-derived organic substances owing to its extraordinarily high phosphorus content. Its discovery drew both attention and criticism at the time, leading to a two-year delay in publishing the results. Notably, this discovery coincided with renowned scientist Gregor Mendel’s breakthrough elucidating the laws of inheritance, marking a pivotal moment in the history of genetics [9].

Miescher quickly recognized that milt, or fish sperm, would make an ideal subject for his research. Milt contained large cells composed primarily of nuclei with minimal cytoplasm and was readily accessible in great amounts. Miescher successfully isolated nuclein from salmon milt harvested from the Rhine River and found that its purity exceeded that of human leukocytes. He used this test to verify that nuclein did not contain sulfur, an impurity likely originating from proteins present in leukocyte samples, as well as to confirm the elevated phosphorus content in nuclein and accurately measure its value. Notably, Miescher observed that all the phosphorus found in nuclein existed as phosphoric acid [10,11]. Simultaneously, his investigations expanded to other species, including carp, frogs, chickens, and bulls. His discovery of nuclein in their sperm samples was remarkable.

Friedrich Miescher was not alone in researching nuclein; other researchers soon responded to his work by developing improved methods for purifying nucleic acids. Richard Altmann, one of Miescher's students and a German pathologist and histologist, believed he had isolated something new due to its acidic chemical reactions. He later named this substance nucleic acid because of this characteristic, not realizing that this was, in fact, what Miescher had referred to as nuclein. In subsequent years, other biologists also made important contributions. Edward Zacharias of Botany made history in 1884 when he demonstrated that nucleic acid is an integral component of chromosomes [12]. The 1893 study of German biochemists Albrecht Kossel and Albert Neumann revealed four bases present in nucleic acid molecules [13]. In addition, Kossel observed nuclein as a part of chromatin, the material that forms chromosomes along with proteins such as histones, which he discovered. Based on these studies, Kossel concluded that nucleic acids have a critical role during the growth and replacement stages [14].

Despite these significant advances, the relevance of nucleic acids remained enigmatic for several decades, and interest in studying them slowly faded until an upsurge in research in the 1930s.

## ESTABLISHING DNA AS THE GENETIC BLUEPRINT: KEY EVIDENCE AND DISCOVERIES

### Chromosome

**Figure 2:**
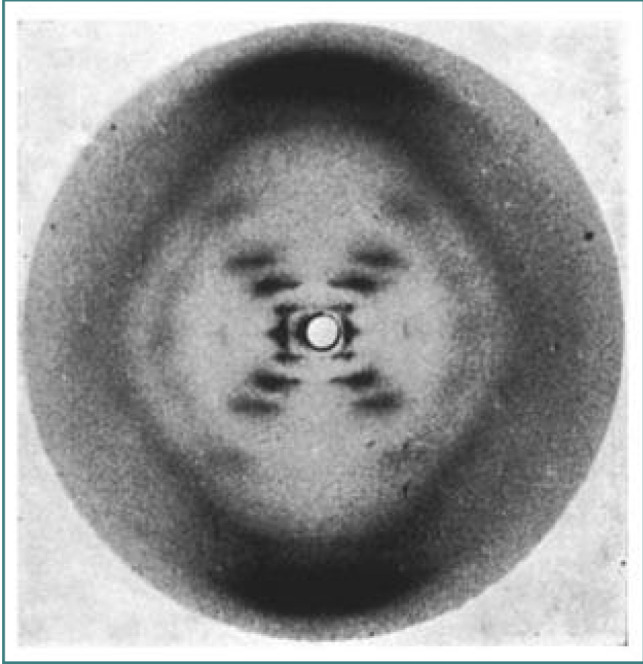
X-ray diffraction image of the double helix structure of the DNA molecule, taken in 1952 by Raymond Gosling during his work with Rosalind Franklin on the structure of DNA, commonly referred to as ‘Photo 51’. © Raymond Gosling/King’s College, London.

As previously discussed, by the beginning of the 20^th^ century, it had already become evident that DNA is a component of chromosomes (Figure 2). The chromosome theory of inheritance was first developed between 1902 and 1904 by German biologist Theodor H. Boveri and American geneticist and physician Walter Sutton, shortly after the rediscovery of Mendel’s laws of inheritance [11–14].

Boveri’s research provided evidence that individual chromosomes within *Paracentrotus lividus* possess distinct characteristics and demonstrated how certain combinations were more essential to proper development than specific numbers. Later, through his investigation of the parasitic nematode *Ascaris megalocephala*, Boveri revealed an essential property known as continuity, which refers to the preservation of genetic material from one generation to the next. Boveri made this observation by monitoring longitudinal changes during mitosis, including those within chromomeres during mitosis and cell division processes. Continuity ensures that cell identity is maintained from cell generation to cell division, which is essential to the continued existence of genetic material [15,16].

Wilhelm von Waldeyer coined the term ‘chromosomes’ years after Theodor Boveri proposed ‘chromatinelemente’ (chromatic elements). This term was adopted and widely used throughout scientific communities worldwide [17].

Walter Sutton, however, conducted studies on the spermatogenesis of the *Brachystola magna* grasshopper [18]. He observed a remarkable parallelism between Mendel’s principles regarding the segregation of character traits during the formation of gametes and the phenomena observed during meiosis. Sutton identified the independent orientation of bivalents during metaphase I of meiosis, which mirrors the independent assortment of distinct character traits as initially described by Mendel. This orientation further substantiates Mendel’s foundational laws of inheritance, specifically the laws of segregation and independent assortment.

In 1929, Hermann J. Muller, Theophilus S. Painter, and Theodosius G. Dobzhansky conducted research that provided direct proof for the chromosome theory of inheritance. They observed that the structural changes to *Drosophila melanogaster* after X-ray exposure were accompanied by changes to its chromosomes, thus providing evidence for correlations between gene order on linkage maps and the physical arrangements of genes on individual chromosomes [19,20].

The discovery of giant chromosomes in *Drosophila* and other dipteran larvae greatly expanded cytogenetical analysis capabilities. Painter used these large chromosomes to produce an elaborate cytological map of *Drosophila melanogaster*'s X chromosome [21]. The bands on this map corresponded with gene blocks found on its linkage map. Painter was able to locate specific genes within certain salivary chromosome bands, providing concrete evidence for Calvin Bridges’s theory of inheritance [22].

### Inheritance DNA

Initially, the concept that chromosomes contain genetic material composed of nucleic acid rather than proteins emerged through mutation studies on various organisms like *Sphaerocarpus donnellii* liverwort, microbial fungi, and maize. Edgar Knapp [23], Alexander Hollaender [24], and Lewis J. Stadler [25] in the United States, along with their colleagues, conducted investigations that revealed a crucial finding: DNA exhibited maximum absorption following exposure to ultraviolet light wavelengths that produced high mutation rates. However, none of these research groups made definitive statements at that time that DNA was the genetic material.

After extensive research, Avery and his colleagues successfully isolated and verified DNA as being responsible for genetic change [26]. Following this discovery, several traits capable of being transferred via DNA in *Pneumococcus* and other bacteria were identified. However, not everyone in the scientific community was convinced. The prevailing belief was that their preparation contained proteins as impurities because proteins were thought to be the only components that could possess the specificity required by genetic material. At that time, Hermann Steudel proposed the tetranucleotide hypothesis, and Phoebus Levene, a Lithuanian-American biochemist, expanded on it further. According to this theory, DNA was thought to consist of identical units of tetranucleotides, each containing one of four bases. However, such an approach seemed too monotonous for a molecule responsible for transmitting genetic information.

Over time, it has become established that, except for viruses, DNA serves as the universal genetic material on this planet. Erwin Chargaff was one of the few scientists who recognized Oswald Avery’s work and acknowledged its validity. Working alongside colleagues in Austria during the late 1940s, Chargaff conducted research that exposed the inaccuracy of the tetranucleotide hypothesis and revealed the specific structure of DNA [27–29]. Notably, Chargaff discovered his signature proportional rule relating to DNA bases; specifically, that they consistently contained equal proportions of adenine (A), thymine (T), guanine (G), and cytosine (C). This finding inspired Watson and Crick’s proposed base-pairing rule as applied to the structure of DNA.

### Ramifications of this groundbreaking discovery

The discovery of DNA has had an indelible impact on medicine. This groundbreaking scientific achievement opened doors to numerous fields that revolutionized our understanding of diseases, diagnostic techniques, therapeutics, and personalized medicine.

For example, the COVID-19 pandemic, caused by the severe acute respiratory syndrome coronavirus 2 (SARS-CoV-2), is one of the greatest public health challenges ever seen worldwide. Genetics and genomics have become essential tools in combatting its spread. Geneticists have had a crucial role in fighting the COVID-19 pandemic by providing insights into the behavior of the virus, tracking its spread, developing diagnostic tests, and informing public health interventions [30].

Genetic sequencing of SARS-CoV-2 has enabled researchers to better understand its evolution and track it over time. By comparing viral genomes from different geographic regions and time points, scientists have been able to detect genetic variants and mutations that may affect transmission, severity, response to treatment regimens, or vaccine efficacy. Genomic surveillance efforts have had a vital role in tracking new variants such as Alpha, Beta, Gamma, and Delta, providing important insight into future vaccine effectiveness or public health strategies.

Moreover, the discovery of the structure of the DNA enabled easier diagnosis of COVID infections. PCR tests targeting specific viral genes such as nucleocapsid gene (N) or spike protein gene (S), have become widely used for detecting active infections. Furthermore, advances in next-generation sequencing technologies have allowed high-throughput sequencing platforms for viral genome surveillance as well as identification of new variants, making these diagnostic tools invaluable in early detection, contact tracing, and tracking virus spread. In addition, Pfizer-BioNTech and Moderna have developed messenger RNA-based vaccines. These vaccines use the genetic information of virus particles to produce fragments that elicit protective immune responses against infection. Genomic surveillance-guided modifications were made to existing vaccines to keep up with emerging variants while maintaining effectiveness [30].

The discovery of the DNA stands out as one of the greatest feats in scientific history, forever changing our view on genetics and life’s building blocks. Its profound implications have transcended disciplines, leading to astounding advancements across various fields of medicine. Neurodegenerative diseases like Alzheimer’s and Parkinson’s have greatly benefitted from DNA discovery as a means of uncovering their intricate mechanisms [31]. These crippling conditions affect millions of individuals worldwide and lead to the progressive impairment of cognitive and motor functions. By exploring the genetic basis of neurodegenerative diseases, we can gain valuable insights that hold great promise for improving diagnosis, treatment strategies, and personalized approaches to combating these difficult conditions. Through DNA, we can embark on a voyage of discovery seeking to decipher its mysteries and forge a path towards improved patient care and better outcomes related to Alzheimer’s and Parkinson’s diseases [32].

## CONCLUSION

The discovery of DNA marked a watershed moment in the history of science, ushering in an age of understanding life itself and unraveling its secrets. This monumental accomplishment stands as one of the crowning achievements in the history of human knowledge. Through relentless scientific enquiry fueled by dedication and passion for discovery, humanity finally acquired ownership of what would become their keystone to existence – the genetic code.

Scientists have unlocked the core of life with the discovery of genetic code. DNA, an elegant molecule that serves as life’s blueprint, holds the key to understanding all living organisms, from microbes to humans alike. As it contains instructions necessary for development, functioning, and perpetuation, it represents an indispensable asset in an uncertain world.

DNA holds meaning beyond mere scientific curiosity; its implications extend far into human society. By understanding and harnessing our genetic code, we gain control over life itself, providing unprecedented advances in medicine, agriculture, biotechnology, and other fields that shape our daily lives. Science offers us a rosy future full of infinite potential. Understanding DNA allows us to dive deeper into heredity mechanisms, uncovering secrets about how traits pass from generation to generation.

This knowledge fuels research on genetic diseases, providing hope for effective treatments and potential cures, while genetically modified crops help provide food security to an ever-increasing global population. Furthermore, advances in personalized medicine allow treatments tailored specifically to an individual’s genetic makeup.

As we delve deeper into scientific inquiry, the journey becomes one of continuous discovery. Knowledge glistens over time and space into eternity itself, yet amidst all this knowledge, an overwhelming sense of awe and reverence for nature’s intricate yet elegant design remains. DNA studies not only reveal the mechanics of life, but also awaken awe for their divine artistry, which sustains all life on this earth.

The discovery of DNA stands as an enduring testament to human perseverance and the never-ending quest for knowledge. With this knowledge at our disposal, humanity embarks on an incredible voyage into an endlessly transformative future, where innovation and understanding possibilities abound. Every step forward opens new windows on eternity while marveling at nature’s magnificent complexity, reminding us all of how interdependent all living things truly are.
